# Molecular Chaperones and Thyroid Cancer

**DOI:** 10.3390/ijms22084196

**Published:** 2021-04-18

**Authors:** Letizia Paladino, Alessandra Maria Vitale, Radha Santonocito, Alessandro Pitruzzella, Calogero Cipolla, Giuseppa Graceffa, Fabio Bucchieri, Everly Conway de Macario, Alberto J. L. Macario, Francesca Rappa

**Affiliations:** 1 Department of Biomedicine, Neuroscience and Advanced Diagnostics (BIND), Institute of Human Anatomy and Histology, University of Palermo, 90127 Palermo, Italy; letizia.paladino@unipa.it (L.P.); alessandra.vitale92@gmail.com (A.M.V.); radha.santonocito@unipa.it (R.S.); alessandro.pitruzzella@unipa.it (A.P.); fabio.bucchieri@unipa.it (F.B.); 2 Euro-Mediterranean Institute of Science and Technology (IEMEST), 90139 Palermo, Italy; AJLMacario@som.umaryland.edu; 3 Consorzio Universitario Caltanissetta, University of Palermo, 93100 Caltanissetta, Italy; 4 Department of Surgical Oncology and Oral Sciences, University of Palermo, 90127 Palermo, Italy; calogero.cipolla@unipa.it (C.C.); giuseppa.graceffa@unipa.it (G.G.); 5 Department of Microbiology and Immunology, School of Medicine, University of Maryland at Baltimore-Institute of Marine and Environmental Technology (IMET), Baltimore, MD 21202, USA; econwaydemacario@som.umaryland.edu

**Keywords:** thyroid tumors, chaperone system, molecular chaperones, chaperonopathies by mistake, Hsp27, Hsp60, Hsp70, Hsp90, chaperonotherapy, differential diagnosis

## Abstract

Thyroid cancers are the most common of the endocrine system malignancies and progress must be made in the areas of differential diagnosis and treatment to improve patient management. Advances in the understanding of carcinogenic mechanisms have occurred in various fronts, including studies of the chaperone system (CS). Components of the CS are found to be quantitatively increased or decreased, and some correlations have been established between the quantitative changes and tumor type, prognosis, and response to treatment. These correlations provide the basis for identifying distinctive patterns useful in differential diagnosis and for planning experiments aiming at elucidating the role of the CS in tumorigenesis. Here, we discuss studies of the CS components in various thyroid cancers (TC). The chaperones belonging to the families of the small heat-shock proteins Hsp70 and Hsp90 and the chaperonin of Group I, Hsp60, have been quantified mostly by immunohistochemistry and Western blot in tumor and normal control tissues and in extracellular vesicles. Distinctive differences were revealed between the various thyroid tumor types. The most frequent finding was an increase in the chaperones, which can be attributed to the augmented need for chaperones the tumor cells have because of their accelerated metabolism, growth, and division rate. Thus, chaperones help the tumor cell rather than protect the patient, exemplifying chaperonopathies by mistake or collaborationism. This highlights the need for research on chaperonotherapy, namely the development of means to eliminate/inhibit pathogenic chaperones.

## 1. Introduction

### 1.1. Thyroid Tumors

Thyroid is an important component of the endocrine system involved in the regulation of basal metabolism, heart rate, blood pressure, and temperature. Thyroid pathologies can cause serious diseases, for example, hyperthyroidism and hypothyroidism. The thyroid disease goiter is characterized by an increase in the gland volume, which can be diffuse or nodular. Although in most cases, the thyroid nodules are benign, they are malignant in a small fraction of patients, representing cases of thyroid cancer (TC).

TC is the most common endocrine cancer. Its incidence has been growing in recent years, especially in females, suggesting that female hormones may be involved in pathogenesis [1]. There are various types of TC, which are classified considering the cell of origin and histological characteristics into two main groups: a) well-differentiated, including papillary thyroid carcinoma (PTC), follicular thyroid carcinoma (FTC), and Hürthle cell carcinoma; and b) poorly differentiated (PDTCs), such as anaplastic/undifferentiated thyroid carcinoma (ATC) and medullary thyroid cancer (MTC). PTC, FTC, and ATC originate from follicular cells, while MTC stems from parafollicular C-cells and constitutes a minor fraction of TC [2,3].

Fine-needle aspiration (FNA) biopsy is the most common diagnostic test in the initial evaluation of patients with a thyroid nodule, yielding a diagnostic accuracy ranging from 70% to 97%. Despite the benefits of FNA cytology for diagnosing papillary, medullary, and anaplastic TC, it is not helpful in determining whether follicular or Hürthle cell thyroid growths are benign or malignant [4]. Therefore, approximately 10% of FNA results lead to misdiagnosis [5]. For this reason, specific genetic alterations, such as proto-oncogene serine/threonine protein kinase (RAF) mutations, have been identified as a reliable marker for improving diagnosis [6]. Other promising biomarkers are members of the chaperone system, whose role in thyroid carcinogenesis is still poorly understood.

### 1.2. The Chaperone System

The main components of the chaperone (also called chaperoning) system are the molecular chaperones, some of which are called heat shock proteins or Hsps [7,8]. They have canonical functions pertinent to maintenance of protein homeostasis that include interactions with the ubiquitin–proteasome system and chaperone-mediated autophagy mechanisms [9,10] and other functions (noncanonical) involving interaction with the immune system with implications in inflammatory and autoimmune disorders, and cancer [11,12,13,14,15,16,17]. Typically, chaperones are cytoprotective but if qualitatively and/or quantitatively abnormal, they can be pathogenetic and cause diseases, namely chaperonopathies [7,12].

Among the chaperonopathies, those by mistake or collaborationism are most relevant to carcinogenesis because cancer cell growth, proliferation, metastasization, and resistance to anticancer drugs may depend on one or more chaperones. These chaperones, apparently qualitatively normal by available methodology but usually quantitatively increased, help the tumor rather than the host, “mistakenly collaborating” with the enemy. Identification of chaperonopathies by mistake as etiologic–pathogenic factors in cancer is key to the development and application of chaperonotherapy, namely the use of chaperones as agents or targets for treatment [18,19]. Therefore, it is pertinent to investigate the role of the chaperone system in thyroid carcinogenesis, beginning by mapping the chaperones in the thyroid tissue and then assessing their quantitative variations in the different tumors at various times during their development.

Here, we will survey illustrative examples of such studies, (Table 1).

## 2. Quantitative Changes of Molecular Chaperones and Associated Effects in TC

Molecular chaperones are involved in the maturation and stabilization of proteins, including those that are oncogenic and, therefore, chaperones are often increased in thyroid cancer tissue. Presumably, this increase is a reflection of the cancer cell’s abnormally high need for chaperones required by its high division rate, rapid growth, and dissemination mechanism, all of which require more functional proteins, correctly folded and assembled than a nonmalignant cell. In the following sections, we will briefly discuss some illustrative examples of studies describing the quantitative patterns of molecular chaperones in TC.

### 2.1. Hsp27

**Figure 1:**
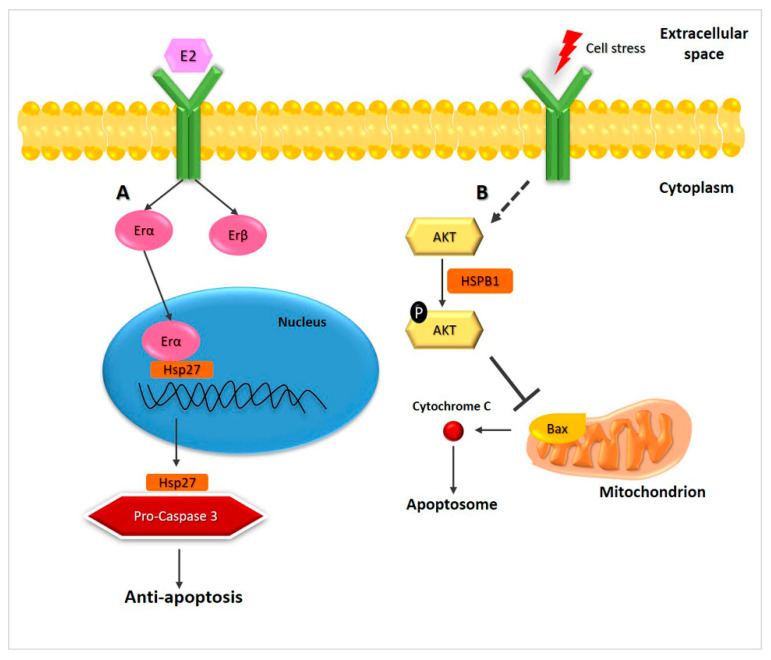
Hsp27 regulates apoptosis. (**A**) Hsp27 can be upregulated by estrogen (E2) levels in human papillary thyroid cancer (PTC) cells that have a higher level of ERα than of ERβ. Overexpression of Hsp27 facilitates proliferation, conferring resistance to apoptosis through interaction with procaspase-3 [29,31]. (**B**) Hsp27(shown as HSPB1), acting as scaffold protein, modulates apoptosis through interaction with Akt. In this way, Hsp27 increases the interaction of Akt with its substrate, Bax, inhibiting Bax activation, oligomerization, and translocation to the mitochondria, thus inhibiting the release of cytochrome C. This prevents the correct formation and function of the apoptosome complex [34,35].

Hsp27 promotes cell survival by inhibiting apoptosis and necrosis, blocking caspase activation [28]. In TC, the Hsp27 increase is correlated with elevated estrogen (E2) levels, which promote growth, invasion, and migration, conferring resistance to apoptosis [29]. In PTC, Erα/SP1 (estrogen receptor/specificity protein 1) can upregulate Hsp27 at the mRNA and protein levels, facilitating proliferation and conferring resistance to apoptosis triggered by TNF-α through interaction with procaspase-3 [29]. Hsp27 interacts with the amino-terminal prodomain of caspase-3, thereby inhibiting the second proteolytic cleavage necessary for caspase-3 activation, Figure 1A [30,31,32]. This mechanism may be the reason why PTC is three times more common in women than in men [33]. Furthermore, in PTC, Hsp27 (HSPB1) is a scaffold protein, which promotes phosphorylation of Akt, inducing cell survival [34]. In this way, Akt, through Bax inhibition, prevents apoptosome formation, Figure 1B [35].

In one illustrative study, the level of Hsp27 was assessed in tumor and nontumor thyroid tissue samples from the same thyroid glands and cultured thyroid cells, by immunohistochemistry and Western blot, respectively [28]. Western blot detected high levels of Hsp27 on different human thyroid cancer cell lines, including the lines K1 (PTC), WRO (FTC), and FRO and KAT18 (ATC). Similarly, immunohistochemical staining showed an increase in the level of antioxidant molecules and Hsp27 in tissue samples from both malignant and benign thyroid tumors, including PTC, FTC, and follicular adenoma (FA), and multinodular goiters, when compared to normal tissue. The same pattern was observed also in human TC cells subjected to oxidative stress [20]. All these data indicate that Hsp27 is elevated in TC cells. Therefore, this chaperone can be considered a candidate biomarker of thyroid malignancy to be monitored in patients.

### 2.2. Hsp60

Although the level of the molecular chaperone Hsp60 in tumor tissues has been studied in various cancers [36,37,38,39], data on thyroid tumors are scarce; Hsp60 could participate in carcinogenesis after undergoing post-translational modifications, such as nitration, acetylation, oxidation, and ubiquitination, which could lead to mitochondrial dysfunction and thereby facilitate tumorigenesis [40].

### 2.3. Hsp70

**Figure 2:**
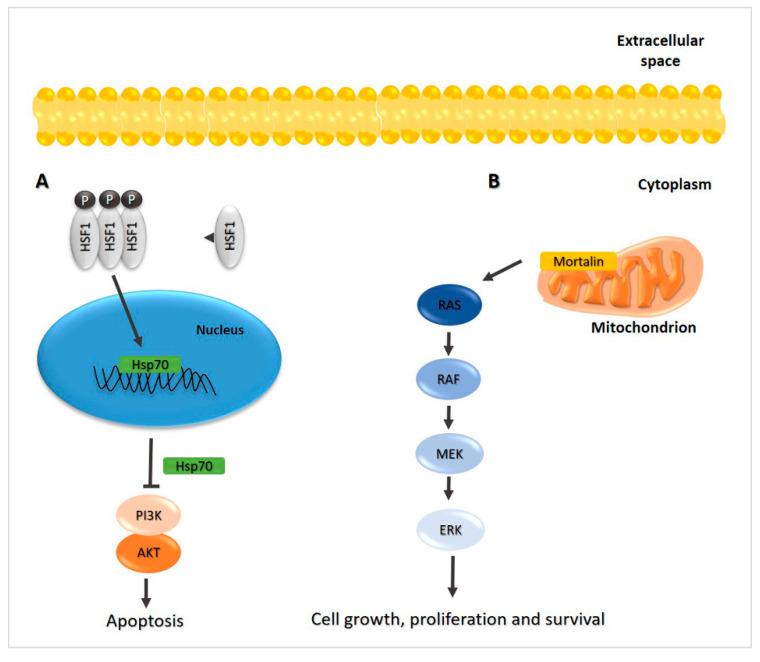
Hsp70 is involved in cancer development at multiple steps. (**A**) Hsp70 induces evasion from apoptosis, blocking PI3K/Akt signaling [43]. (**B**) Mortalin (also called HSPA9 or GRP75), localized in mitochondria, alters MEK/ERK signaling, ensuring cell survival [23].

Hsp70 levels are elevated in a variety of tumors, often correlated with tumor grade, metastases, and poor prognosis [41,42]. Members of the Hsp70 family play a variety of roles in the maintenance of protein homeostasis and, therefore, at least some of them must participate in carcinogenesis and can be used as targets for anticancer drugs. For example, VER155008 is a selective Hsc70 inhibitor that induces death of ATC cells, mediated through PI3K/Akt signaling, Figure 2A [32,43].

Mortalin (HSPA9/GRP75) plays a role in MTC cell proliferation and survival and its upregulation is associated with bad prognosis and chemoradiotherapy resistance, as is also the case for other tumors [25]. Mortalin is localized in mitochondria and its depletion is accompanied by depolarization of the mitochondrial membrane, ROS (reactive oxygen species) generation, and apoptotic cell death. Mortalin depletion induces not only cell cycle arrest by altering MEK/ERK signaling but also induces caspase-dependent apoptotic cell death, Figure 2B. These data suggest that mortalin could be a target for treatment, since it is a key regulator of cell signaling and metabolism in MTC [23,32].

Western blot showed elevated levels of mortalin in human MTC, TT, and MZ-CRC-1 cell lines when compared with normal human fibroblasts [23]. In addition, it was revealed by immunohistochemistry that mortalin was increased in human MTC, PTC, FTC, and ATC compared to normal thyroid tissues, suggesting a possible role of the chaperones in carcinogenesis, probably related to its role in maintaining the homeostasis of the proteins involved in mitochondrial bioenergetics and redox balance [23,24]. Mortalin depletion induced cell death and growth arrest in different human cell lines, including PTC (PTC-1 cell line), FTC (FTC133 cell line), and ATC (8505C, and C643 cell lines), and in mouse xenografts, revealing its role in promoting tumor cell survival and proliferation, which prompted the proposal of mortalin as therapeutic target [23,24].

Immunohistochemistry, immunocytochemistry, and immunoblotting were used to assess the levels of the estrogen receptor alpha36 (ERα36), GRP78, and GRP94 in PTC specimens [26]. Their levels were elevated in primary PTC tissues and derived cells, while no follicular cells showed positive ERα36, GRP78, or GRP94 immunostaining in normal thyroid tissues and nodular hyperplasia tissues [26]. Their high levels were found to be strongly associated with aggressive PTC, suggesting these three proteins as possible indicators of PTC aggressiveness measured by estimating extra-thyroid extension (ETE), lymph node metastasis (LNM), distant metastasis (DM), and high tumor node metastasis (TNM) stage [26]. Moreover, it was found that the levels of GRP78 and GRP94 are increased by estrogen E2 [26] as found also in other cancers [44,45,46,47], suggesting a role of E2 in the progression and metastasis of PTC.

The levels of GRP78 assessed by immunostaining in primary tumor and noncancerous thyroid tissues were found significantly higher in MTC specimens than in normal controls, while the levels of Hsp70 and Hsp90 were also elevated in cancerous tissues but not significantly [25].

### 2.4. Hsp90

**Figure 3:**
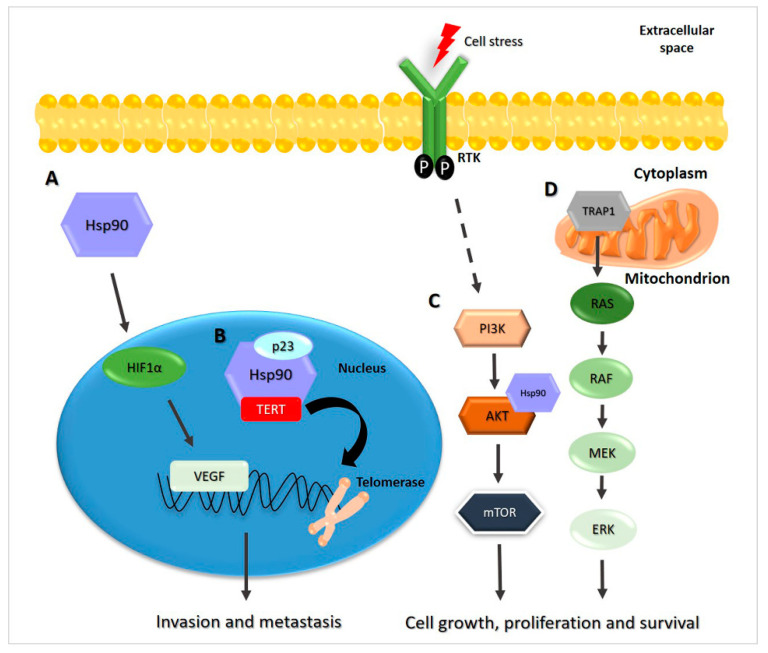
Hsp90 is involved in cancer development in pathways critical for the cell growth, invasiveness, and survival. Hsp90 regulates the hypoxia-inducible factor (HIF)-1α activity [50] (**A**), and together with chaperone p23, binds the human telomerase reverse transcriptase (hTERT) [54] (**B**), causing evasion from apoptosis and metastasis. (**C**) The components of the PI3K/AKT/mTOR pathway are Hsp90 clients, indicating the chaperone’s role in the regulation of autophagy. Therefore, autophagy has a pro-survival function [32,49]. (**D**) The tumor necrosis factor receptor-associated protein 1 (TRAP1) is involved in TCs: it is thought to enhance ERK phosphorylation and cell cycle progression, leading to a dampening of apoptosis by activation of the RAS/RAF/ERK pathway [56].

Hsp90 levels in TC tumor tissue have been found elevated by comparison with normal peritumoral tissue [48,49]. Generally, Hsp90 increased levels correlated with a poor prognosis [50,51,52]. Hsp90 is involved in the activities of β-catenin, BRAF, AKT, survivin, and some members of the Bcl-2 (B-cell lymphoma 2) complex [32]. Thus, inhibition of Hsp90 induces tumor cell apoptosis, lessening migration and invasion, Figure 3C [49].

Hsp90 regulates hypoxia-inducible factor (HIF)-1α activity, suggesting that it might have a role in PTC tumorigenesis, Figure 3A [50]. Hsp90 regulates telomerase activity in TC [53]. Probably, Hsp90, together with chaperone p23, binds and activates the human telomerase reverse transcriptase (hTERT) [54]. This activation could be an important step in tumorigenesis since the cells gain the ability to proliferate indefinitely and become immortal [55]. For this reason, increased Hsp90 levels and telomerase activity have been described as markers that correlated with malignancy and tumor progression [53,54]. Likewise, it has been shown that levels of p23 and Hsp90 mRNAs were elevated in cancerous thyroid tissue by comparison with non-malignant thyroid tissue, Figure 3B [53].

Another member of the Hsp90 family, the TRAP1 (tumor necrosis factor receptor-associated protein 1) chaperone, is also involved in protection from apoptosis, drug resistance, and cell cycle progression [56]. TRAP1 is increased in various human malignancies, including TC, with its levels progressively increasing from normal peritumoral thyroid tissue to PTC tissue [57]. The involvement of TRAP1 in TC occurs via modulation of ERK phosphorylation and cell-cycle progression, leading to increased apoptosis by constitutive activation of the RAS/RAF/ERK pathway, Figure 3D [32,56].

Because various proteins necessary for thyroid cancer growth are Hsp90 clients, inhibition of the chaperone seems a promising anticancer therapy. For instance, the compounds 17-allylamino-17-demethoxygeldanamycin, KU711, and WGA-TA are Hsp90 inhibitors that could interrupt oncogenic pathways in TC [32,58,59]. A natural product, BTIMNP_D004, was evaluated for anticancer activity in the following TC cell lines: papillary cancer cell lines (BCPAP and TPC1), follicular cancer cell lines (FTC133, FTC236, FTC238), medullary cancer cell line (DRO81-1), anaplastic cancer cell line (SWI736), and a fibroblast cell line (MRC-5) as control. The colorimetric assay MTS was used for measuring cell viability/proliferation and evaluating the effect of BTIMNP_D004. It was observed that BTIMNP_D004 inhibited the proliferation TC cells more effectively than the proliferation of fibroblasts. Levels of Hsp90 were evaluated by Western blot and showed that in FTC133 cells treated with the drug, Hsp90α increased whereas Hsp90β decreased, but in BCPAP cells, the levels of Hsp90α decreased. Likewise, it was demonstrated that the levels of ERK decreased in BCPAP cells, and the levels of AKT decreased in FTC133 cells [60].

Another two Hsp90 inhibitors, KU711 and WGA-TA, were tested in the TC cell lines TPC1, FTC238, WRO, and ACT1 [59]. Migration and invasion assays, with the use of Boyden chambers and matrigel chambers, respectively, were conducted to test the effect of KU711 and WGA-TA on the cell lines, and it was found that the drug caused a decrease in migration and invasion. Western blot analyses of Hsp90 and its client proteins BRAF and AKT in TC cell lines treated with the drugs were also conducted. The results showed that the levels of Hsp90 were stable compared with the control, whereas the levels of AKT and BRAF decreased [59].

Immunoblot analysis to assess the levels of TRAP1 and the client proteins BRAF and ERK, with or without TRAP1 silencing, was conducted using the FTC cell lines ML1 and FTC133, the PTC cell line BCPAP, and the ATC cell lines BHT101 and CAL62. This study showed that TRAP1 silencing inhibited both BRAF and ERK pathways in TC [56].

## 3. Various Chaperones Assessed Simultaneously

Recently, our research group has estimated the levels of Hsp27, Hsp60, Hsp70, and Hsp90 in thyroid cancerous tissues and adjacent peritumoral tissues from patients with FA or FTC by immunohistochemistry. The level of Hsp27 was high but did not show a significant quantitative variation in cancerous tissues compared to the corresponding normal parenchyma, whereas Hsp60, Hsp70, and Hsp90 were increased in FTC compared to FA and/or to adjacent normal parenchyma [22]. In another study, the immunohistochemical levels of Hsp27, Hsp60, and Hsp90, but not Hsp70, were high in PTC as compared with benign goiters and normal peritumoral tissues [21]. Both works also reported the intracellular distribution of each Hsp, showing not only an increased level in cancerous compared to normal tissues, but also an altered localization, with accumulation in the cytoplasm and plasma cell membrane in cancerous specimens [21,22]. These results agree with other works showing a change in the cellular distribution or even extracellular secretion of Hsps during carcinogenesis processes [61,62,63,64,65].

A comprehensive immunomorphological description of the distribution of molecular chaperones in TC enhances the visualization of the malignant patterns and constitutes one of the benefits of immunohistochemistry [21,22]. For example, immunohistochemistry was used to evaluate the levels of Hsp70 and Ki-67 (a proliferation marker) and their subcellular localization in relation to clinical and morphological parameters of PTC [66]. The immunohistochemical detection of Hsp70 showed that it was differentially localized in tumor cells (cytoplasm, nucleus, nucleolus, or a combination of these different sites), and that its nuclear translocation was correlated with the stage of the carcinogenic process and with prognosis: the nuclear translocation of the protein was found higher in samples with stage IV and with an unfavorable prognosis.

## 4. Exosomes and TC

Almost all cell types release extracellular vesicles (EVs), such as exosomes, including cancer cells [67,68]. It is assumed that exosomes released by cancer cells can interact with cells around the tumor, both cancerous and stromal, and with distant cells through the circulation, possibly promoting cancer development. The latter could be mediated by one or more of the following mechanisms: enhancement of cell proliferation and invasiveness, stimulation of angiogenesis, induction of immune suppression, and remodeling of the tumor microenvironment to favor metastasis [69,70,71]. These mechanisms and effects are still under investigation. Despite the current uncertainties, circulating EVs and their molecular cargo can be considered a promising source of biomarkers for the early detection, diagnosis, monitoring, and follow up of different types of cancer. Unfortunately, unlike other tumors for which there is considerable information, such as glioblastoma and prostate, lung, ovarian, and breast cancers [72,73,74,75,76,77,78], only two studies have been reported on exosomal molecular chaperones in TCs and their potential applications [21,27]. The levels of Hsp27, Hsp60, and Hsp90 were increased in the exosomes from the plasma of PTC patients compared with the same patients after thyroidectomy and patients with benign goiter [21]. In addition, it was demonstrated that Hsp27 was increased in exosomes from the plasma of PTC patients with lymph node metastases, suggesting the potential of this molecular chaperone as an indicator of lymphatic metastasis [27].

## 5. Conclusions and Perspectives for the Future

The results discussed in the preceding paragraphs show that quantification of chaperones by immunohistochemistry can be useful for differential diagnosis and to follow their changes in relation to the evolution of the disease. Furthermore, immunohistochemistry provides information on the distribution of the chaperones inside and outside the cells with the potential for revealing patterns that distinguish different tumors from each other. In addition, the data suggest that chaperones may play an etiologic–pathogenic role and encourages research aimed at elucidating the molecular mechanisms underpinning the quantitative changes of the chaperones and their effects on the tumor cell.

Tumor classification has historically been based on the primary anatomic site in which the tumor occurs and on its morphologic and histologic phenotypes. However, histopathology alone cannot accurately predict the prognosis and treatment response of individual cancers [79]. It is necessary to quantify morphological and molecular features of cells and tissues under physiological and pathological conditions, noting that each cell unit possesses the nucleus and cytoplasm, which structurally and functionally cooperate for gene expression and cellular dynamics [80]. For this reason, molecular and cell morphological methods have increased in number and versatility in recent years. In this review, we discuss studies of the quantitative profiles of chaperones in thyroid tissue, of cancerous compared to normal. The results provide an incentive to progress toward elucidation of the molecular mechanisms underpinning the observed quantitative variations. By what mechanism do chaperones augment (or decrease) in cancer cells? Likewise, the following question arises: what role, if any, do chaperones play in tumor initiation and progression? It is possible that the changes observed in the levels of chaperones in thyroid tissue, particularly those indicating an increase in quantity of chaperone molecules inside the tumor cells, are the consequence of the special needs of the cancer cell with its high proliferation rate and metabolism in addition to its dissemination tendency. Higher quantities of correctly folded proteins and their functional assemblages are required by cancer cells as compared to normal ones, which call for higher quantities of chaperones. Thus, chaperones do play a role in carcinogenesis by assisting cancer cells so they can successfully perform key functions characteristic of malignancy, which they could not perform adequately if chaperones were scarce. Thyroid cancer, at least some of its forms, can therefore be considered chaperonopathies by mistake or collaborationism. A normal chaperone—normal in so far as can be determined by current technology—mistakenly helps, so to speak, the enemy; it collaborates with the enemy rather than the contrary. The importance of this concept resides in the fact that it alerts physicians and researchers to the possibility of developing/applying chaperonotherapy, namely the use of therapeutic strategies and compounds targeting the mistaken chaperones. This strategy is exemplified by studies with Hsp90 inhibitors, which have been proposed as anticancer drugs in different forms of TCs since inhibition of the chaperone causes the simultaneous degradation of multiple proteins that are Hsp90 clients involved in oncogenic signaling pathways [17]. For instance, the novel non-geldanamycin Hsp90 inhibitor NVP-AUY922 induced apoptosis in PTC cells and death by disrupting the complex Hsp90/survivin, thus causing the downregulation of survivin [49]. Likewise, Hsp90 inhibition has been proposed to enhance the effect of other anticancer drugs [43].

## Figures and Tables

**Table 1:** Molecular chaperone levels in thyroid tumors.

	Chaperone	Tumor	Quantitative Level	Reference
**Immunohistochemistry**	Hsp27 (HSPB1)	FA ^1^	Increased	[20]
		FTC	Increased	[20]
		PTC	Increased	[21]
	Hsp60	FTC	Increased	[22]
		PTC	Increased	[21]
	Hsp70	FTC	Increased	[22]
	Mortalin (HSPA9; GRP75)	ATC	Increased	[23,24]
		FTC	Increased	
		MTC	Increased	
		PTC	Increased	
	GRP78	MTC	Increased	[25]
		PTC	Increased	[26]
	GRP94	PTC	Increased	[26]
	Hsp90	FTC	Increased	[22]
		PTC	Increased	[21]
**Biochemistry; immunochemistry**	Hsp27	ATC	Increased	[20]
		FTC	Increased	
		PTC	Increased	
	Mortalin (HSPA9; GRP75)	MTC	Increased	[23]
	GRP78	PTC	Increased	[26]
	GRP94	PTC	Increased	[26]
**Exosomes**	Hsp27	PTC	Increased	[21,27]
	Hsp60	PTC	Increased	[21]
	Hsp90	PTC	Increased	[21]

^1^ Abbreviations: FA, follicular adenoma; ATC, anaplastic thyroid carcinoma; FTC, follicular thyroid carcinoma; MTC, medullary thyroid cancer; PTC, papillary thyroid carcinoma.

## Abbreviations

AKT	serine/threonine protein kinase B
ATC	anaplastic/undifferentiated thyroid carcinoma
Bcl-2	B-cell lymphoma 2
DM	distant metastasis
ERK	extracellular signal-regulated kinases
Erα/SP1	estrogen receptor/specificity protein 1
ERα36	estrogen receptor alpha36
ETE	extra-thyroid extension
EVs	extracellular vesicles
FA	follicular adenoma
FNA	fine-needle aspiration
FTC	follicular thyroid carcinoma
(HIF)-1α	hypoxia-inducible factor-1α
hTERT	human telomerase reverse transcriptase
LNM	lymph node metastasis
MEK	mitogen-activated protein kinase kinase
MTC	medullary thyroid carcinoma
mTOR	mammalian target of rapamycin
PDTC	poorly differentiated thyroid carcinoma
PI3K	phosphatidylinositol 3-kinase
PTC	papillary thyroid carcinoma
RAF	proto-oncogene serine/threonine protein kinase
ROS	reactive oxygen species
TC	thyroid cancer (or thyroid carcinoma)
TNM	tumor node metastasis stage
TRAP1	tumor necrosis factor receptor-associated protein 1
